# Changing HIV treatment eligibility under health system constraints in sub-Saharan Africa: investment needs, population health gains, and cost-effectiveness

**DOI:** 10.1097/QAD.0000000000001190

**Published:** 2016-09-07

**Authors:** Jan A.C. Hontelez, Angela Y. Chang, Osondu Ogbuoji, Sake J. de Vlas, Till Bärnighausen, Rifat Atun

**Affiliations:** a Harvard T.H. Chan School of Public Health, Harvard University, Boston, Massachusetts, USA; b Department of Public Health, Erasmus MC, University Medical Center Rotterdam, Rotterdam, The Netherlands; c Africa Centre for Population Health, University of KwaZulu-Natal, Mtubatuba, South Africa.

**Keywords:** cost-effectiveness, health system constraints, HIV prevention, HIV treatment, investment needs, mathematical modeling, sub-Saharan Africa

## Abstract

**Objective:**

We estimated the investment needs, population health gains, and cost-effectiveness of different policy options for scaling-up prevention and treatment of HIV in the 10 countries that currently comprise 80% of all people living with HIV in sub-Saharan Africa (Ethiopia, Kenya, Malawi, Mozambique, Nigeria, South Africa, Tanzania, Uganda, Zambia, and Zimbabwe).

**Design:**

We adapted the established STDSIM model to capture the health system dynamics: demand-side and supply-side constraints in the delivery of antiretroviral treatment (ART).

**Methods:**

We compared different scenarios of supply-side (i.e. health system capacity) and demand-side (i.e. health seeking behavior) constraints, and determined the impact of changing guidelines to ART eligibility at any CD4^+^ cell count within these constraints.

**Results:**

Continuing current scale-up would require US$178 billion by 2050. Changing guidelines to ART at any CD4^+^ cell count is cost-effective under all constraints tested in the model, especially in demand-side constrained health systems because earlier initiation prevents loss-to-follow-up of patients not yet eligible. Changing guidelines under current demand-side constraints would avert 1.8 million infections at US$208 per life-year saved.

**Conclusion:**

Treatment eligibility at any CD4^+^ cell count would be cost-effective, even under health system constraints. Excessive loss-to-follow-up and mortality in patients not eligible for treatment can be avoided by changing guidelines in demand-side constrained systems. The financial obligation for sustaining the AIDS response in sub-Saharan Africa over the next 35 years is substantial and requires strong, long-term commitment of policy-makers and donors to continue to allocate substantial parts of their budgets.

## Abstract 2

Supplemental Digital Content is available in the text

## Introduction

The fight against HIV in sub-Saharan Africa (SSA) has a strong momentum with the scale-up of treatment and prevention interventions. The number of people receiving antiretroviral treatment (ART) has increased from less than 100 000 in 2004 to almost 12 million in 2015 [1], vastly improving life-expectancy of the general population [2]. Yet, despite these impressive achievements, the HIV epidemic is still very much an unfinished agenda. By 2015, the number of people living with HIV in the world had risen to 37 million, of which 70% were in SSA [1]. ART provision in SSA is still suboptimal due to constraints on both the demand-side (i.e. the population level demand for HIV care) and the supply-side (i.e. the capacity of the health system to meet population need) [3,4]. HIV incidence in SSA remains high [5], and the success of ART means increasing numbers of people require long-term treatment and care [6,7]. Long-term financial commitments through both donor contributions and increased domestic funding of treatment programs are therefore critical if the fight against HIV is to be sustained and further expanded [8–11].

Because ART not only extends and improves the lives of HIV-infected people, but also substantially reduces their infectiousness [12,13], expanding access to treatment will benefit both the HIV-infected and HIV-uninfected populations [14–17]. The WHO released guidelines for HIV treatment in mid-2013, recommending threshold for ART initiation to be raised from CD4^+^ cell counts of 350 cells/μl or less to 500 cells/μl or less [17]; and in 2015 the WHO released updated treatment guidelines (http://www.who.int/hiv/pub/arv/policy-brief-arv-2015/en/), recommending that ART should be initiated at any CD4^+^ cell count. Providing ART at any CD4^+^ cell count could simplify HIV care delivery in SSA, as triaging according to CD4^+^ cell count or disease stage is no longer needed, and the relatively high rates of loss-to-follow-up and associated mortality in pre-ART care could potentially be avoided [18]. In addition, the Strategic Timing of Antiretroviral Treatment (START) trial recently showed that initiation of ART at CD4^+^ cell counts of more than 500 cells/μl results in health benefits for the individual patient [19].

Mathematical modeling can assist policy-makers in SSA and donors globally to better understand future obligations and the impact of future funding for HIV and thereby make informed decisions in budget allocation and ART treatment guidelines development. We have recently provided estimates of the investment needs for the HIV in nine SSA countries and shown that there are still substantial funding gaps in place [20], raising questions on the optimal policy choices in ART scale-up in countries faced with health system constraints. Therefore, a comprehensive evaluation of the cost-effectiveness and resource needs of different policy options is needed – both on tackling supply-side and demand-side constraints as well as changing guidelines to treat HIV-infected people at any CD4^+^ cell count within existing constraints.

We used the established STDSIM model [7,16,21–23], and expanded the model for this study to capture the underlying dynamics of demand-side and supply-side interactions of ART delivery that ultimately determine ART coverage, to estimate the future investment needs, population health gains, and cost-effectiveness for a wide range of policy options for the AIDS response in Ethiopia, Kenya, Malawi, Mozambique, Nigeria, South Africa, Tanzania, Uganda, Zambia, and Zimbabwe. These countries account for about 80% of the current HIV burden in SSA [5]. We compared different scenarios of supply-side and demand-side constraints in ART delivery and determined the impact of changing ART eligibility at any CD4^+^ cell count for these scenarios.

## Methods

STDSIM is a stochastic microsimulation model that simulates individuals in a dynamic network of sexual contacts [16]. The model incorporates health system constraints on both the supply-side and demand-side of HIV prevention, treatment, and care, allowing for more realistic projections of the effects of different ART treatment scale-up scenarios. We used previously published country level quantifications for the 10 countries [7], and we updated and refined these country level fits to accurately reflect updated epidemiological and ART uptake data [5].

### Model and fitting

In the model, HIV is described in four consecutive stages with exponentially distributed durations: early infection, asymptomatic infection, symptomatic infection, and AIDS. Average survival of an individual with untreated HIV is about 10 years [24]. ART treatment uptake in the model is the result of two submodels. The first represents an individual's demand for ART as a function of disease stage, whereas the second describes the health systems capacity to meet population need [25].

For the purpose of this study, we updated and refined previously published country quantifications of STDSIM for Ethiopia, Kenya, Malawi, Mozambique, Nigeria, South Africa, Tanzania, Uganda, Zambia, and Zimbabwe [7]. A more detailed description of the model, our approach, and the resulting fit of model projections to data are described in the Supplementary material.

### Scenarios

We modeled six scenarios reflecting pessimistic (scenarios 1 and 2), realistic (scenarios 3), and optimistic (scenarios 4, 5 and 6) future health system developments. Supply-side constraints are progressively removed in scenarios 1 to 4 keeping demand-side constraints intact; whereas demand-side and supply-side constraints are removed in scenarios 5 and 6. For each scenario, we modeled two ART eligibility thresholds (ART at CD4^+^ cell counts of 500 cells/μl or less and ART at any CD4^+^ cell count). The scenarios are as follows:

1. *Current financing obligations* – continue treating patients who are currently on treatment; no other people are initiated on treatment as of 1 January 2016 (*pessimistic scenario*).
2. *Health system constraints* – the number of people on ART as of 1 January 2016 is maintained; people can only initiate treatment when slots free-up because of mortality or loss-to-follow-up (*pessimistic scenario – supply-side and demand-side constraints*).
3. *Continued scale-up* – current rate of treatment scale-up continues into the future until all supply-side constraints are removed (*realistic scenario – demand-side constraints*).
4. *Rapid scale-up* – All supply-side constraints are removed per 1 January 2016 (*optimistic scenario – demand-side constraints*).
5. *90-90-90*; target set by The Joint United Nations Programme on HIV/AIDS (UNAIDS) [26] – all supply-side constraints are removed per 1 January 2016 annual population-based screening for HIV at 90% coverage removes demand-side constraints. All HIV-infected people participating in screening are linked to care and initiated on ART when eligible (*optimistic scenario – no constraints*).
6. *Expanded 90-90-90 (90-90-90+)* – scale-up of the following prevention services next to the 90-90-90 scenario (*optimistic scenario – no constraints*): male circumcision (80% coverage), condom distribution (non-use reduced by 60%), behavior change (overall partner change rates reduced by 10%), and prevention of mother-to-child transmission (95% coverage). Details are in Supplementary material Section 2.5.

### Costs

We derived unit costs for treatment-related services by combining data from the multicountry analysis of treatment costs for HIV/AIDS study [27] and Menzies *et al.*[28]. We set the cost of first-line antiretrovirals cost at US$132 per person per year (2012 USD) [27], and second-line antiretrovirals at US$366 (2013 USD) [29]. All ART treatment costs were further increased by 20% to account for above facility programmatic costs [30]. We determined costs from the healthcare provider perspective, and did not include medical costs incurred offsite (i.e. inpatient days), patient time, and patient travel costs. Cost-effectiveness of strategies and scenarios was calculated as the cost in US$ per life-year saved, and we applied traditional willingness to pay threshold values in terms of Gross Domestic Product (GDP) per capita income.

Rapidly scaling up ART treatment access by relaxing supply-side constraints will require upfront investments in health system improvements. Therefore, we calculated a once-off cost reflecting health system improvements when needed, with data derived from National AIDS Spending Assessment country reports [31]. More details on all cost assumptions are given in the Supplementary material Section 3 and Table S5.

## Results

**Fig. 1:**
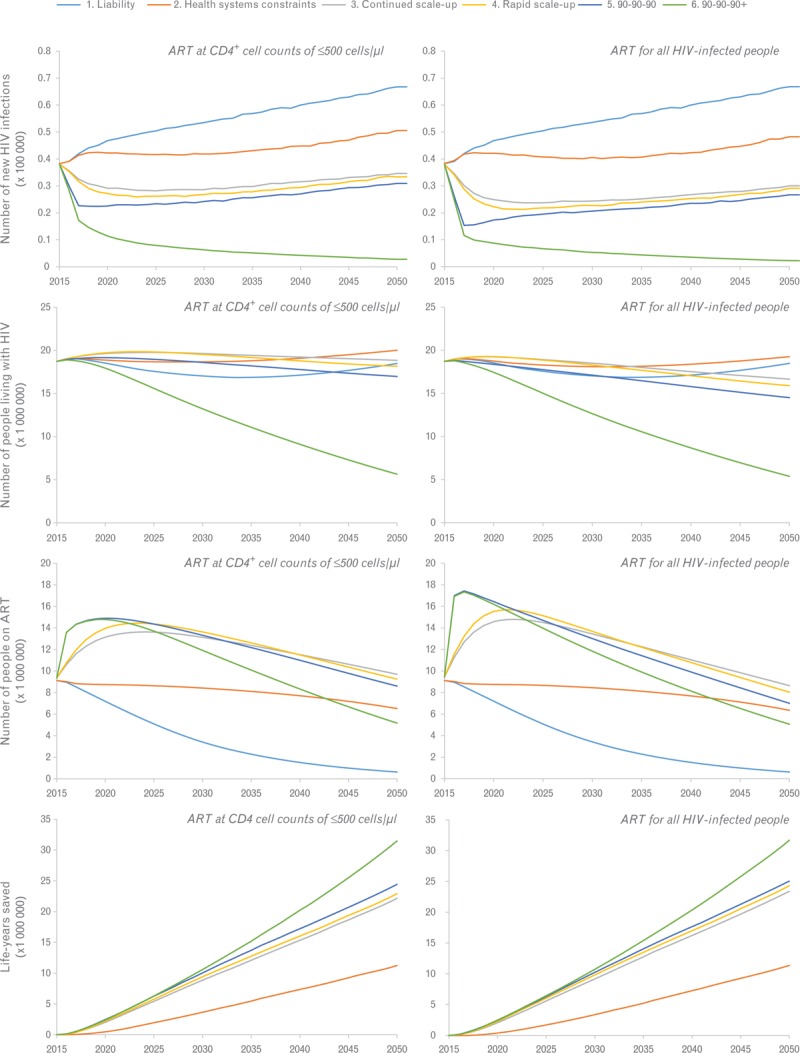
Predicted trends in number of people living with HIV, new HIV infections, people on antiretroviral treatment, and life-years saved (compared with the liability scenario) over the period 2015–2050 for the 10 countries with the largest HIV epidemics in sub-Saharan Africa.

Figure 1 shows the predicted number of people living with HIV, new HIV infections, people on ART, and life-years saved in the 10 countries for the six scenarios under guidelines of ART eligibility at CD4^+^ cell counts of 500 cells/μl or less (left panels) and under ART eligibility at any CD4^+^ cell count (right panels). The optimistic 90-90-90+ scenario provides the largest impact on incidence: from 320 000 per year to only 27 000 (under ART at CD4^+^ cell counts of 500 cells/μl or less) and 22 000 new infections (under ART at any CD4^+^ cell count). Because demand-side constraints limit the number of people initiating treatment with high CD4^+^ cell counts, the number of people on treatment when offering ART at any CD4^+^ cell count is only slightly higher compared with ART eligibility at CD4^+^ cell counts of 500 cells/μl or less in scenarios 1–4.

The HIV burden in 2015 was divided over the 10 countries as follows: 31% in South Africa, 16% in Nigeria, 8% in Uganda, 8% in Mozambique, 7% in Tanzania, 7% in Kenya, 7% in Zimbabwe, 5% in Zambia, and 3% in Ethiopia.

**Fig. 2:**
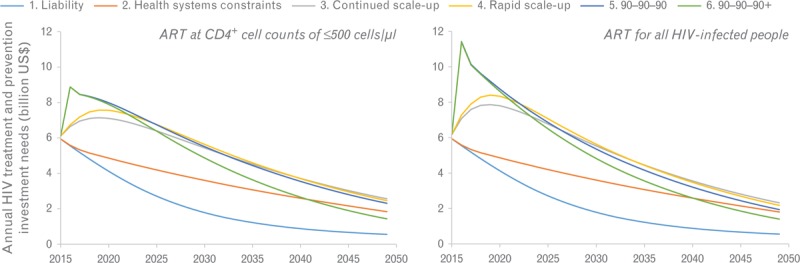
Average annual HIV treatment and prevention costs in the 10 countries with the largest HIV epidemic in sub-Saharan Africa.

Annual investment needs peak at about US$11 billion for the expanded 90-90-90 scenario under ART eligibility at any CD4^+^ cell count and are between US$6 and US$8 billion for continued (scenario 3) and rapid scale-up (scenario 4) of health systems access (Fig. 2).

Costs include antiretroviral treatment costs, circumcision, condom distribution, and prevention of mother-to-child transmission (PMTCT) screening and treatment for Ethiopia, Kenya, Malawi, Mozambique, Nigeria, South Africa, Tanzania, Uganda, Zambia, and Zimbabwe. Costs and effects are discounted at an annual rate of 3%. Treatment costs represent about 80% of all costs, followed by PMTCT (12%), male circumcision (5%), condom distribution (2%), and behavior change (1%).

### Removing heath system constraints

Cumulative total investment needs, population health gains, and cost-effectiveness (compared with scenario 1) when removing health system constraints are shown in Table 1. Continued scale-up of ART with demand-side constraints remaining in place (scenario 3) would need an investment of US$178 billion and would result in about 11 million infections averted and 377 million life-years saved by 2050 (ICER: 272 US$/life-year saved). Removing both supply-side and demand-side constraints (scenario 5) would need an investment of US$186 billion, resulting 13 million infections averted and 424 million life-years saved. The most cost-effective option is to also scale-up HIV prevention interventions (scenario 6) with slightly lower investment needs compared with scenario 5 (US$165 billion) yet more infections averted (about 20 million) and life-years saved (about 493 million).

### Changing eligibility guidelines

**Fig. 3:**
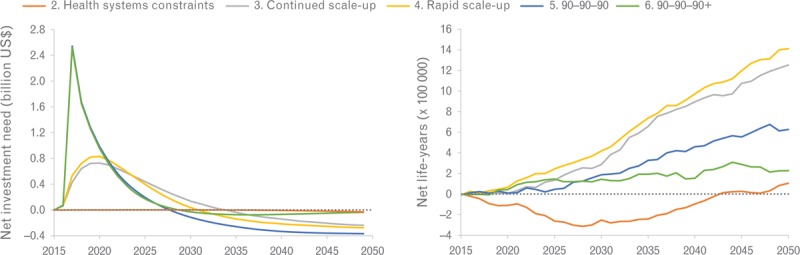
Incremental costs and life-years of changing guidelines from antiretroviral treatment at CD4^+^ cell counts of 500 cells/μl or less to antiretroviral treatment at any CD4^+^ cell count in the 10 countries with the largest HIV epidemic in sub-Saharan Africa, for five scenarios.

The annual incremental investment needs and life-years saved of changing guidelines from ART at CD4^+^ cell counts of 500 cells/μl or less to ART at any CD4^+^ cell count within each scenario of health system constraints are shown in Fig. 3. The incremental investment needs for changing guidelines are highest in the 90-90-90 and 90-90-90+ scenarios (about US$1.6 billion each year), whereas in the continued scale-up scenario, only relatively modest upfront investments of about US$700 million annually are needed, an increase of about 10%.

Guidelines are changed to HIV infected people at any CD4^+^ cell count, within each health system constraint scenario in 2016. The liability scenario is not included since no new people will be initiated on antiretroviral treatment after 2016 in this scenario, so a guidelines change will have no net effects. Costs and effects are discounted at an annual rate of 3%.

Importantly, changing guidelines in scenarios in which demand-side constraints are still in place (realistic scenarios) will result in more life-years saved (about 1.2 and 1.4 million additional life-years saved for continued and rapid scale-up, respectively) compared with the optimistic scenarios in which supply-side and demand-side constraints are removed completely (0.2 to 0.6 million life-years saved for 90-90-90 and 90-90-90+ scenarios, respectively) (Fig. 3). This effect occurs because refusing people treatment at CD4^+^ cell counts of more than 500 cells/μl in demand-side constrained systems will often result in patients defaulting pre-ART care and subsequent late presentation or mortality. These detrimental effects are prevented when people are initiated at any CD4^+^ cell count. In a system with no constraints (e.g., the 90-90-90 scenario), these people will still be initiated as soon as their CD4^+^ cell counts drop below 500 cells/μl, making the incremental benefit of initiation at any CD4^+^ cell count less profound.

The cost-effectiveness of changing eligibility to ART at any CD4^+^ cell count ranges from US$54 per life-year saved under health systems constraints to US$1358 per life-year saved for the 90-90-90+ scenario (Table 2). Even under our most conservative willingness to pay threshold, changing guidelines is cost-effective in all scenarios except for the 90-90-90+ scenario. Nevertheless, changing guidelines under severe supply-side constraints would result in a loss of about 4.6 million life-years because healthier patients are crowding out patients with more advanced disease. Alternative assumptions on health-seeking behavior had a limited impact on these results (Table S6).

## Discussion

Continuation of the AIDS response in SSA over the period 2016–2050 will require substantial investment needs. Under eligibility criteria of ART at CD4^+^ cell counts of 500 cells/μl or less, annual investment needs between 2016 and 2020 in the 10 countries that share 80% of the HIV burden in SSA range from about US$5 billion in the undesirable pessimistic scenario of only treating patients currently on ART to about US$7–8 billion if health system access is continuously improved. Total investment needs through to 2050 under continued scale-up would amount to about US$180 billion. Changing guidelines to provide ART at any CD4^+^ cell count is highly cost-effective in all scenarios, especially in scenarios with demand-side constraints. However, providing ART at any CD4^+^ cell count under supply-side constraints will result in population health loss, as healthy patients are crowding out people with advanced disease.

To our knowledge, this study is the first that shows the effects of changing guidelines to treating HIV-infected people at any CD4^+^ cell count within realistic scenarios of health systems that are experiencing supply-side and demand-side constraints. Previous modeling studies have shown that universal test and treat can substantially reduce HIV incidence [14,16,32], yet these studies all relied on optimistic scenarios of high levels of treatment uptake, similar to our 90-90-90 scenarios. We show that changing eligibility guidelines to ART at any CD4^+^ cell count in a health system with demand-side constraints has more benefits (about 20 million life-years saved) compared with scenarios of universal test and treat (5 to 10 million life-years saved), whereas initial investment needs are only a modest 10% higher compared ART eligibility at CD4^+^ cell counts of 500 cells/μl or less.

There are currently several large-scale community randomized trials underway that examine the feasibility and impact of the concept of treatment as prevention in a real-world settings [33,34]. Although these studies are valuable in assessing the potential impact and feasibility of a universal test and treat approach [35], we show that health system constraints may provide sufficient ground to change guidelines. Triaging HIV treatment is becoming increasingly complex, with different considerations by CD4^+^ cell count, HIV disease stage, pregnancy status, coinfections with tuberculosis or hepatitis, and the HIV status of a cohabiting sexual partner. Providing treatment at any CD4^+^ cell count will substantially simplify treatment delivery in settings with weak health systems and thus improve efficiency. In addition, high rates of loss-to-follow-up in patients that are referred to pre-ART care because their HIV status does not match with eligibility criteria could be avoided. Furthermore, the START trial showed that earlier treatment initiation would also result in improved treatment outcomes in individual patients as well [19].

Regardless of changes in guidelines and future scenarios, our results show that continued fight against HIV will require substantial investments over the coming decades. However, the economic climate is challenging, and international funding for HIV is flat lining [36]. Innovative funding sources must be considered [37], along with improvements in health system efficiency and better allocation of available resources to meet future financing obligations.

Our results show that scaling up prevention interventions along with expansion of ART is highly beneficial. The added value of increased rates of circumcision, condom use, prevention of mother-to-child transmission (PMTCT), and lower rates of partner change in our 90-9090 scenario is about 6 million averted adult infections, 800 000 averted child infections, and 70 million life-years saved by 2050, and about US$300 million of cost-savings. Although there is an ethical obligation to provide treatment for all HIV-infected patients in need, our results present a strong case of continuous investments in other prevention interventions alongside treatment scale-up.

Our long-term predictions are subject to the assumption that future transmission dynamics, interventions, and other factors will remain the same. However, it is likely that this will not be the case. Our study did not include the investment needs and population health gains of a PrEP roll-out in SSA because of limited available data on potential strategies and scenarios. However, PrEP may well be added to the mix of HIV prevention in SSA in the near future [38]. In addition, there have been substantial advances in search of an HIV vaccine and cure [38], and future discoveries might affect the long-term HIV response. Further, other unforeseeable dynamics – such as large-scale behavior changes, or the general economic, demographic, and social context in SSA might influence future HIV transmission dynamics, making long-term model predictions necessarily imprecise. Nevertheless, these factors will likely influence all scenarios in our analysis in a similar fashion and may therefore not change our main conclusions substantially.

Some simplifications in our study should be taken into account. First, due to a lack of data, unit costs in our analysis were assumed to follow linear functions according to program scale-up. This might not be the case in real life, as scaling-up large-scale programs is subject to economies of scale in which per patient costs decrease as fixed costs of a program are spread across progressively more patients [39,40]. Further, the cost of antiretroviral drugs could potentially further decline in the future [41]. We also did not account for the efficiency benefits of removing triaging and pre-ART care from ART delivery because limited availability of data prevented us from incorporating these effects. Contrastingly, it may also be possible that reaching additional patients is more costly and less efficient because they are harder to reach and are likely to perform poorest in treatment programs, potentially offsetting efficiency gains because of scale effects and declining unit costs. Second, we applied a simple additional cost reflecting increased health worker training and infrastructural development needed to reduce supply-side constraints. As the required personnel might not be readily available and requires years of training, short-term staff increases may also require diverting healthcare workers from other services or pulling them from the private sector, requiring additional funding. Due to limited data availability, we were unable to incorporate these costs and may therefore have slightly underestimated the costs for health system improvements in the first years of our projections, yet this will have limited impact on our overall projections. In addition, scarcity of human resources for health might limit short-term implementation of more optimistic scenarios entirely [42] and require innovative interventions such as task-shifting of community-based service delivery. Third, we could not take potential negative outcomes of changing guidelines, such as poorer adherence of healthier patients or transmission of drug resistant strains, into account due to lack of available data. Fourth, we did not include below-facility costs incurred by patients through patient time and patient travel costs [43], yet the main focus of our study was to inform public-sector spending decisions. Fifth, some of our scenarios might be unrealistic. Especially our most pessimistic scenario, in which no new patients initiate treatment, is unlikely to happen in any country. However, we think that comparing our more realistic scenarios with this pessimistic scenario illustrates both the continued investment needs as well as the health benefits of continuing the fight against HIV. Furthermore, immediate implementation of our most optimistic scenarios seems unlikely, as resource constraints would require more gradual scale-up. Sixth, supply-side and demand-side constraints in our model only affect the total number of people on ART and do not reflect the potential effects on reduced rates of viral suppression among those on ART.

We considered an international benchmark on the basis of GDP per capita to define scenarios as (highly) cost-effective. However, we realize that this benchmark is poorly grounded in economic theory and therefore somewhat arbitrary [44]. In addition, it might also be ethically moot to let the valuation of human life depend on per capita GDP. Nevertheless, we still consider it convenient in the absence of local comparative cost-effectiveness information. In addition, we compared cost-effectiveness to several willingness to pay thresholds, including a very conservative threshold, and our conclusions were robust to these assumptions.

There have been other efforts to quantify the future investment needs, of which the most notable are AIDS2031, the ‘HIV Investment Framework’ Study Group, and our recent estimates of investment needs in nine countries [20,29,45]. The projections from these studies are all based on the Goals model and could not comprehensively incorporate the underlying dynamics that determine the uptake of ART nor provide estimates on the costs and effects ART scale-up and guideline changes within the context of existing constraints. We used the comprehensive microsimulation model STDSIM, which dynamically captures HIV transmission and the effects of interventions. In addition, rather than assigning a coverage level of ART treatment to a certain population at a certain point in time, our model is able to capture the underlying dynamics of demand-side and supply-side interactions of ART delivery that ultimately determine ART coverage. This allows us to model the impact of different scenarios of health system constraints on the future developments of ART coverage instead of making rather arbitrary assumptions on how coverage might change in the future.

In conclusion, we show that changing guidelines to ART eligibility at any CD4^+^ cell count could be a cost-effective policy option, even when ART delivery is subject to health system constraints. Especially in demand-side constrained system, abandoning triaging of ART delivery could improve efficiency and help avoid the excessive loss-to-follow-up and mortality in patients receiving pre-ART care. Next to ART scale-up, investment in other prevention interventions is a relatively low-cost commitment that could translate into cost-savings. However, regardless of eligibility guidelines for ART and scale-up scenarios, the investment needs for the AIDS response in SSA over the next 35 years are substantial and require strong, long-term commitments of policy-makers and donors to continue to allocate substantial parts of their budgets to treatment and prevention of HIV.

## Acknowledgements

The current article was published as part of RethinkHIV. RethinkHIV is a consortium of senior researchers, funded by the RUSH Foundation, who evaluate new evidence related to the costs, benefits, effects, fiscal implications, and developmental impacts of HIV interventions in sub-Saharan Africa. Jan A.C. Hontelez is a Netherlands Organisation for Scientific Research (NWO) Talent Scheme Veni fellow. J.A.C.H., A.Y.C., O.O., and R.A. designed the analysis. Cost data were derived from literature by A.Y.C. J.A.C.H. performed all the analysis. J.A.C.H. and R.A. wrote the initial draft of the manuscript. All authors assisted in interpreting the results and writing the final draft of the manuscript.

Source of funding: This article was published as part of RethinkHIV. RethinkHIV is a consortium of senior researchers, funded by the RUSH Foundation, who evaluate new evidence related to the costs, benefits, effects, fiscal implications, and developmental impacts of HIV interventions in sub-Saharan Africa. Jan A.C. Hontelez is a Netherlands Organisation for Scientific Research (NWO) Talent Scheme Veni fellow.

### Conflicts of interest

There are no conflicts of interest.

## Supplementary Material

Supplemental Digital Content

## Figures and Tables

**Table 1:** Cumulative total and incremental investment needs, population health gains, and cost-effectiveness over the period 2016–2050 for incrementally removing demand-side and supply-side constraints under different HIV treatment guidelines.

	Investment need		Population health gains						Cost-effectiveness
	Total (billion US$) (range)	Incremental^a^ (billion US$) (range)	New infections (millions) (range)		Infections averted^a^ (millions) (range)		Population life-years (billions) (range)	Life-years saved^a^ (millions) (range)	ICER (cost in US$ per life year saved) (range)
			*Horizontal transmission*	*Vertical transmission*	*Horizontal transmission*	*Vertical transmission*			
ART eligibility at CD4^+^ cell count ≤500 cells/μl									
1. Liability	76 (67; 86)	n.a.	19.3 (14.0; 25.4)	3.2 (2.3; 4.3)	n.a.	n.a.	17.9 (17.2; 18.6)	n.a.	n.a.
2. Health systems constraints	125 (113; 137)	49 (47; 51)	15.4 (10.7; 20.9)	2.4 (1.6; 3.3)	4.0 (3.3; 4.4)	0.8 (0.7; 0.9)	18.1 (17.4; 18.8)	174 (168; 180)	284 (280; 286)
3. Continued scale-up	178 (158; 201)	103 (91; 116)	10.8 (7.5; 14.4)	1.2 (0.8; 1.7)	8.5 (6.4; 10.9)	2.0 (1.5; 2.6)	18.3 (17.6; 19.0)	377 (363; 390)	272 (252; 296)
4. Rapid scale-up	183 (163; 206)	108 (97; 120)	10.1 (7.1; 13.9)	1.1 (0.7; 1.5)	9.2 (6.8; 11.5)	2.2 (1.6; 2.8)	18.3 (17.6; 19.0)	395 (380; 409)	273 (255; 295)
5. 90-90-90	186 (166; 208)	110 (99; 123)	9.1 (6.3; 12.4)	0.9 (0.6; 1.3)	10.3 (7.7; 13.0)	2.3 (1.7; 3.0)	18.4 (17.7; 19.0)	424 (408; 439)	260 (243; 279)
6. 90-90-90+	165 (149; 180)	88 (82; 94)	2.5 (1.8; 3.3)	0.1 (0.1; 0.2)	16.8 (12.1; 22.0)	3.1 (2.2; 4.1)	18.4 (17.8; 19.1)	493 (475; 511)	180 (173; 185)
ART eligibility at any CD4^+^ cell count									
1. Liability	76 (67; 86)	n.a.	19.3 (14.0; 25.4)	3.2 (2.3; 4.3)	n.a.	n.a.	17.9 (17.2; 18.6)	n.a.	n.a.
2. Health systems constraints	125 (113; 137)	49 (47; 51)	14.8 (10.3; 20.1)	2.4 (1.6; 3.2)	4.5 (3.7; 5.3)	0.9 (0.7; 1.0)	18.1 (17.4; 18.8)	169 (164; 175)	290 (288; 293)
3. Continued scale-up	182 (161; 204)	106 (95; 119)	9.2 (6.6; 12.3)	1.0 (0.7; 0.13)	10.1 (7.4; 13.1)	2.3 (1.6; 2.9)	18.3 (17.7; 19.0)	396 (381; 411)	269 (249; 289)
4. Rapid scale-up	186 (165; 209)	110 (99; 124)	8.6 (6.1; 11.6)	0.9 (0.6; 1.2)	10.7 (7.8; 13.8)	2.3 (1.7; 3.1)	18.4 (17.7; 19.0)	417 (402; 432)	264 (246; 286)
5. 90-90-90	188 (169; 207)	112 (103; 122)	7.6 (5.4; 9.9)	0.8 (0.5; 1.0)	11.8 (8.6; 15.5)	2.5 (1.8; 3.2)	18.4 (17.7; 19.0)	434 (417; 449)	259 (246; 271)
6. 90-90-90+	172 (156; 188)	96 (89; 102)	2.0 (1.5; 2.7)	0.1 (0.1; 0.1)	17.3 (12.5; 22.7)	3.2 (2.3; 4.2)	18.4 (17.8; 19.1)	499 (480; 517)	193 (186; 197)

Net health gains and costs are compared with the liability scenario, ranges are shown between brackets and reflect uncertainty in model fit parameters (see Supplementary material for more details). Horizontal infections represent sexually transmitted HIV, vertical infections reflect mother-to-child transmission of HIV. ART, antiretroviral treatment; ICER, incremental cost-effectiveness ratio; n.a, not applicable.

^a^Incremental to the liability scenario.

**Table 2:** Investment needs, population health gains, and cost-effectiveness of changing antiretroviral treatment treatment guidelines from antiretroviral treatment at CD4^+^ cell counts of 500 cells/μl or less to antiretroviral treatment for all HIV-infected people – in the 10 countries with the largest HIV epidemics in sub-Saharan Africa.

		ART eligibility at any CD4^+^ cell count vs. ART eligibility at CD4^+^ cell count ≤500 cells/μl				
Scenario	Constraints	Investment needs	Population health gains			Cost-effectiveness
		Incremental cost (million US$) (range)	Infections averted (million) (range)		Life-years saved (million) (range)	ICER (costs in US$ per life year saved) (range)
			*Horizontal transmission*	*Vertical transmission*		
2. Health systems constraints	Supply-side and demand-side (*pessimistic*)	−246 (−224; −271)	0.5 (0.4; 0.8)	0.1 (0.1; 0.1)	−4.6 (−4.7; −4.4)	54 (51; 57)
3. Continued scale-up	Only demand-side (*realistic*)	3979 (3530; 4496)	1.6 (1.0; 2.2)	0.2 (0.1; 0.3)	19.2 (18.4; 19.8)	208 (191; 227)
4. Rapid scale-up	Only demand-side (*optimistic*)	2555 (2276; 2877)	1.5 (1.0; 2.3)	0.2 (0.1; 0.3)	22.4 (21.5; 23.2)	114 (106; 124)
5. 90-90-90	No constraints (*optimistic*)	2184 (1950; 2451)	1.5 (0.9; 2.5)	0.1 (0.1; 0.2)	10.0 (9.6; 10.3)	219 (203; 237)
6. 90-90-90+	None (*optimistic*)	7463 (6747; 8165)	0.5 (0.4; 0.6)	0.1 (0.0; 0.1)	5.5 (5.3; 5.7)	1358 (1276; 1435)

Ranges are shown between brackets and reflect uncertainty in model fit parameters (see Supplementary material for more details). Horizontal infections represent sexually transmitted HIV, vertical infections reflect mother-to-child transmission of HIV. The liability scenario is not shown, because in this pessimistic scenario ART is only continued for all currently receiving ART, and there are no new imitations after 2016. Hence, changing guidelines to ART at any CD4^+^ cell count would have no effect on costs and population health. ART, antiretroviral treatment; ICER, incremental cost-effectiveness ratio.
