# Supplementary material for: Changing HIV treatment eligibility under health system constraints in sub-Saharan Africa: investment needs, population health gains, and cost-effectiveness
Source: AIDS. 2016 Sep 7;30(15):2341–50. doi: 10.1097/QAD.0000000000001190 (PMC5017264; doi:10.1097/QAD.0000000000001190)
Supplement: Supplemental Digital Content [file aids-30-2341-s001.docx]

**Supplementary material to manuscript:**

**Changing HIV treatment eligibility under health system constraints in sub-Saharan Africa: Investment needs, population health gains, and cost-effectiveness**

Authors: Jan A.C. Hontelez,^1,2,3^ Angela Y. Chang,^1^ Osondu Ogbuoji,^1^ Sake J de Vlas,^2^ Till Bärnighausen,^1,3^ Rifat Atun^1^

1. Harvard T. H. Chan School of Public Health, Harvard University, Boston, USA

2. Department of Public Health, Erasmus MC, University Medical Center Rotterdam, Rotterdam, Netherlands

3. Africa Centre for Health and Population Studies, University of KwaZulu-Natal, Mtubatuba, South Africa

Contents

[**1.** **General model structure** 3](#_Toc419113011)

[**2.** **Country specific quantifications** 4](#_Toc419113012)

[**2.1 Original country quantifications** 4](#_Toc419113013)

[**2.2 Sexual behaviour and risk profiles** 4](#_Toc419113014)

[**2.3 Updated country quantifications** 6](#_Toc419113015)

[**2.4 Model fit to data** 7](#_Toc419113016)

[**2.5 Other prevention interventions** 7](#_Toc419113017)

[**3.** **Unit costs** 8](#_Toc419113018)

[**4.** **Simulations** 8](#_Toc419113019)

[**5.** **References** 16](#_Toc419113020)

1. **General model structure**

We used STDSIM, a stochastic microsimulation model of the transmission and control of HIV and other sexually transmitted infections (STIs) [1-4]. The model simulates the life course of individuals in a dynamic network of sexual contacts. Events like partnership formation or the acquisition of infections are the result of random processes, determined by probability distributions. Therefore, the results of the model are subject to stochastic variation.

The model consists of four modules: *demography*, *sexual behaviour*, *transmission and natural history*, and *interventions*. The demography module implements the processes of birth, death, and migration. Processes for initiation and dissolution of sexual relationships, for mixing according to age preference, for sexual contacts within relationships and for sexual contacts between clients and sex workers are defined in the sexual behaviour module. In the transmission and natural history module, transmission probabilities per sexual contact are specified for HIV and other simulated STIs. Finally, the interventions module specifies the timing and effectiveness of control measures in curbing transmission or enhancing survival.

In the model, HIV is described in 4 consecutive stages, with exponentially distributed durations: early infection (average of 10 weeks); asymptomatic infection (average of 5.5 years); symptomatic infection (average of 4 years); and AIDS (average of 0.75 years). Average survival of an individual with untreated HIV is about 10 years [5]. We assume ART to reduce infectiousness of HIV by 90% [6]. New insights from observational studies in South Africa [7] show that the survival benefits of ART are much more substantial than previously estimated [8]. The estimated life-expectancy of HIV infected people starting ART at CD4 counts of <200 cells/μL (weighted average of 32 years for people aged 20) [7] is about 16 times higher compared to untreated patients with CD4 <200 cells/μL (mean of 2 years) [9]. We therefore assumed the average duration of HIV stages to be a factor 16 longer for those receiving ART compared to those not receiving ART.

ART treatment uptake in the model is the result of 2 sub-models. The first represents an individual’s demand for ART as a function of disease stage, while the second describes the health systems capacity to meet population need. Rates of seeking care are assumed to increase during disease progression, as HIV-infected people experiencing symptoms are more likely to seek care.

Demand-side constraints are the result of sub-optimal health seeking behavior, in which individuals seek care at a very late stage or not at all. As a result of these constraints, patients who are deemed to be not eligible for treatment after an initial test for eligibility may also only return for re-testing of CD4 counts late or not at all. Health seeking behavior as a function of diseases stage has previously been quantified for rural South Africa using empirical data from the Africa Centre for Population Health in KwaZulu-Natal [1]. Because extensive sensitivity analyses on health seeking in previous studies showed a limited impact of different rates on model outcome [1], we assume the same rates to be applicable for the other countries in this study. In addition, we performed a sensitivity analysis on the main outcome of our study, the cost-effectiveness of ART at any CD4 cell count versus ART at CD4 cell count ≤500 cells/µL under all scenarios of constraints, by increasing and decreasing health seeking rates by 20% (results are presented in table S6).

Supply-side constraints are modelled as a suboptimal probability of individuals who seek care to be initiated on treatment, reflecting constraints in the health care system to provide ART due to infrastructural, logistic, or human resource limitations.

Model runs start in the year 1910 with a fixed population size. The country-specific background age-specific fertility and mortality rates create a population of about 20,000 to 50,000 in 2014. This modelled population size is extrapolated to the observed population sizes in the 10 countries. In order to do this, we divide the population size in 2014 as reported by the United Nations World Population Prospects [10] by the modelled population size in 2014, and apply the obtained ratio to all years. HIV is introduced in the population by randomly infecting 2 FSWs at a user-defined (country specific) year of introduction.

Further details about the general model structure can be found in previous publications [2-4, 11, 12]. The modeling of antiretroviral therapy (ART) is described by Hontelez *et al* [1, 11, 13].

1. **Country specific quantifications**

For the purpose of this study, we updated the country specific quantifications for Ethiopia, Kenya, Malawi, Mozambique, Nigeria, South Africa, Tanzania, Uganda, Zambia, and Zimbabwe that were previously used to study the impact of ART on the age composition of the HIV epidemic [14]. The original quantifications were developed to roughly reproduce HIV prevalence and ART treatment scale-up of all countries in SSA over the period 2000 to 2009. We now fine-tuned the quantifications for the 10 countries listed above to reproduce the observed HIV prevalence and ART treatment scale-up over the period 1990 to 2013. Our approach is described below.

## **2.1 Original country quantifications**

The original quantifications used in Hontelez *et al* [14] resulted from a standardized approach in which we ran the model with country-specific mortality, fertility, and circumcision rates, and epidemic specific sexual behavior profiles for each country in SSA. Background mortality rates (mortality in the absence of HIV) were calculated using country-specific life tables [15], and burden of disease estimates published by the World Health Organization (WHO) [16]. For each country, we first calculated the proportion of deaths attributed to HIV through comparison of the age- and sex-specific burden of disease estimates [16], and the all-cause mortality rates in the WHO life tables [15]. We then used the ratio between these two mortality estimates (HIV-specific and all-cause) to compute background mortality rates for all causes except for HIV. Age- and period-specific fertility rates for each country were obtained from the 2008 United Nations (UN) World Fertility Data [17]. We assumed that fertility rates remained constant after 2011. We obtained country-specific circumcision prevalence from literature [18, 19].

## **2.2 Sexual behaviour and risk profiles**

For each of country, we selected one of three sexual behaviour profiles that describes the age at sexual debut, time until availability, age-specific rates of relationship formation and contact frequencies, durations of relationships, age-mixing, and commercial sex. Sexual behaviour profiles were obtained from the 4-cities study, and were named according to the epidemics they produced: *Concentrated profile*; *mixed profile*; and *generalized profile* [14]. Three of the four parameter settings for relationship formation and mixing of the 'four cities study' fitted these three profiles, and were chosen accordingly: Cotonou, Benin (*concentrated risk profile*); Yaoundé, Cameroon (*mixed risk profile*); and Kisumu, Kenya (*generalized risk profile*) [3]. Table S1 gives an overview of the profile selection and circumcision prevalence for the 10 countries in the current analysis. We give a more detailed description on the mechanisms of partnership formation and sexual behaviour in STDSIM below, and more details about the original country quantifications and parameter values of the sexual behaviour profiles can be found in Hontelez *et al* [14].

The model contains three types of sexual relationships: steady relationships, casual relationships, and commercial sex. The formation of partnerships occurs according to a supply- and demand-based mechanism. People become available for a sexual relationship at an *age of sexual debut*, which is randomly drawn at birth from a uniform distribution. Each time the partnership status of a person changes (e.g. a partnership is formed or ended), a new duration until the person becomes available for a new relationship (*time until availability*) is drawn from a predefined exponential distribution with µ being the mean *time until availability* defined as: $\mu= \tau_{s,r}/(r_{s,a}\times p)$

With: $\tau_{s,r}$ = time interval by person's sex (*s*) and relationship status (*r*)

$r_{s,a}$= specific promiscuity factor by sex (*s*) and age (*a*)

*p* = personal promiscuity level

The *personal promiscuity factor* (*p*) reflects the heterogeneity in the tendency to form partnerships between individuals, and is given by a gamma distribution with an average value (*p_m_*) of 1.0, and a shape parameter of 1.5 [3].

The duration of the *availability period* of an individual is given by an exponential distribution, with mean *time to find* (κ) defined as:$\frac{\delta}{r_{s,a} \times p}$ , where the δ is an average duration of the availability period [2]. $r_{s,a}$ and *p* are explained above. When a person is available for a new relationship, he/she can be selected by an individual of the opposite sex who has ended his/her *availability period*. If a person is not selected at the end of the *availability period*, he/she will select a partner from the pool of available persons of the opposite sex. The type of relationship (steady or casual) that is formed when a partner is selected depends on the age of the male partner, and is defined as a *probability of a steady relationship*. The probability of a new relationship being a casual relationship is given by 1 - *probability of a steady relationship*. A relationship starts with a sexual contact. After each contact, the time until a new sexual contact qwithin the relationship is drawn from an exponential distribution with a mean *frequency of sexual contact* depending on relationship type and the age of the male partner. Finally, the duration of a new relationship is drawn from an exponential distribution, where the *average relationship duration* is depends on the relationship type.

Partner selection at the end of the *time to find* is guided through an age preference matrix, which defines the probability of selecting a partner from a certain age class. When there is no partner available in the preferred age class, immediate re-sampling is done of a new preferred age-class using the remaining age groups with a probability larger than 0.0. If no partner can be found in any of the age-classes, a new *time to find* is drawn from the above described equation. Probabilities in the age-preference matrix are chosen to have men on average prefer slightly younger women.

In the model, male clients can visit female sex workers (FSW). A male’s frequency of FSW visit is determined by defining frequency classes (e.g. 0, 1, and 12 times per year [14]). For each class, the proportion of men with and without a steady relationship falling in that category can be specified. A personal prostitute visiting inclination (ranging from 0 to 1, assigned to each male at birth) determines which individual males are assigned to which frequency classes. At sexual debut and at each FSW visit, the next FSW visit is scheduled according to an exponential distribution with the mean duration until next visit is based on the FSW visit frequency of the individual. The number of FSWs in the model results from the male demand. New FSWs are recruited from sexually active females with a defined age range. The number of available FSWs and their predefined number of clients per week is checked each year and matched with the number of visitors. If the number FSWs is too low, new FSWs are recruited. If the number is too high, a random selection terminates their career.

|  | **Sexual behaviour profile** | **Circumcision prevalence** |
| --- | --- | --- |
| Ethiopia | Concentrated | 76% |
| Kenya | Mixed | 84% |
| Malawi | Mixed | 27% |
| Mozambique | Generalized | 56% |
| Nigeria | Concentrated | 81% |
| South Africa | Generalized | 35% |
| Tanzania | Mixed | 70% |
| Uganda | Mixed | 25% |
| Zambia | Mixed | 12% |
| Zimbabwe | Generalized | 10% |

**Table S1. Sexual behaviour profiles and circumcision prevalence for 10 countries in SSA.** Sexual behaviour profiles are described in detail in Hontelez et al [14]

## **2.3 Updated country quantifications**

We updated and refined the original country quantifications described in section 2.1 for the purpose of the current study to reproduce HIV prevalence and ART treatment scale-up over the period 1990-2014. We developed a range of acceptable HIV prevalence and ART coverage estimates for each year by dividing the ‘high estimate’ and ‘low estimate’ of the total number of adult (aged 15 years and over) people living with HIV as reported by UAIDS [20], by the total annual population size as reported by the UN [21].

For each country, we divided the epidemic into 2 phases: 1) the rapid increase of the epidemic; and 2) the levelling off or decline of the epidemic. All previously defined parameters on demography (see section 2.1) and relationship formation and sexual mixing (see section 2.2.1) were kept the same for all 10 countries [14]. We used 2 parameters – year of HIV introduction and overall partner change rates (multiplier of personal promiscuity *p*; see section 2.2.1) – to fit the first phase of the epidemic for all countries. We randomly drew combinations of these parameters from a uniform distributions, and accepted parameter combinations if the simulated HIV prevalence fell between the ‘high estimate’ and ‘low estimate’ of UNAIDS reported HIV prevalence in the population aged 15 years or older from 1990 until the end of the growth phase of the epidemic. As Ethiopia and Nigeria were countries that had the *concentrated risk profile*, we also allowed the personal inclination of men to visit sex workers (see section 2.2.1) to vary by a maximum of 25% up or down for these countries, similar to our approach in Hontelez *et al* [14] .

Next, we fitted ART coverage over the period 2004 - 2013 to the coverage levels reported by UNAIDS [20], using two sub-models. The first sub-model represents an individual's demand for ART as a function of HIV-disease stage; the second sub-model describes the capacity of the health system to meet this demand [11, 13, 14, 22]. ART coverage in our model is the ART demand met by the capacity of the health system. To fit the modelled ART coverage to the annual coverage data reported by UNAIDS (for the period 2004-2013 [20]), we used a scale-up of ART capacity in the health system, while assuming the ART demand function to be the same as previously estimated for South Africa [22]. For each country, we randomly draw the start year slope of the ART treatment scale-up, and accept parameter combinations when simulated ART treatment coverage fell between the ‘low estimate’ and ‘high estimate’ for 2013, and at least 2/3^rd^ of all years between 2004 and 2012.

Finally, we fitted the second phase of the country specific epidemics by increasing rates of condom use to explain the levelling off or decline of the epidemic [23-25]. For each country, we randomly draw a start year, slope, and end value of condom use in casual relations and commercial sex, and accept parameter combinations if the simulated HIV prevalence fell between the ‘high estimate’ and ‘low estimate’ of UNAIDS reported HIV prevalence in the population aged 15 years or older over the period 1990 – 2013. Baseline condom use in casual relationships and commercial sex for all countries was 10%.

We repeated all steps described above until we arrived at 40 unique parameter combinations for each country that described the HIV prevalence and ART scale-up. Results are subsequently calculated by averaging the predictions of the 40 parameter combinations to develop point estimates, and 95% intervals around the estimate are obtained by using the 2^nd^ lowest and 2^nd^ highest values.

## **2.4 Model fit to data**

Figure S1 shows the simulated HIV prevalence in the 10 countries compared to the UNAIDS reported HIV prevalence over the period 1990-2014. For all countries, the model was able to accurately reproduce the UNAIDS prevalence estimates. Figure S2 shows the simulated ART treatment coverage compared to data. The parameter values for all countries are given in Table S2 and Table S3.

## **2.5 Other prevention interventions**

We simulate the following prevention interventions: male circumcision, condom use, PMTCT, and behavior change. Table S4 gives an overview of the HIV prevention services for the different scenarios. STDSIM does not explicitly model mother to child transmission of HIV. For each 5-year age group of women between 15 and 49, we obtained the number of women not on ART from the model, and multiplied this by the annual fertility rate for each age-group to estimate the annual number of women needing PMTCT for that age group. We multiply this need with a country specific coverage level to arrive at the PMTCT resource utilization. Values for prevention targets in the 90-90-90+ scenarios were derived from the UNAIDS defined targets: PMTCT coverage of 95%; condom non-use reduced by 60%; circumcision of young adults (aged 14-25) scaled up to 80% (for countries with a circumcision prevalence below 80%); partner change rates reduced by reducing the personal promiscuity factor (*p – see section 2.1.1*) by 10%. All interventions are scaled-up linearly from 2016 and reach their targets in 2020.

To estimate the effects of PMTCT in reducing the number of pediatric infections, we assumed a transmission rate of 22.5% for untreated women [26], and 1.5% for women on PMTCT [27].

1. **Unit costs**

We derived unit costs for treatment-related services by combining data from the MATCH study (Ethiopia, Malawi, Rwanda, South Africa, Zambia) [28] and Menzies et al. (Botswana, Ethiopia, Nigeria, Uganda) [29]. We regressed the non-ARV costs by GDP per capita income of study countries to derive the linear relationship that allowed us to estimate the non-ARV costs for other countries. Non-ARV costs include opportunistic infection medications, laboratory costs, nutritional support, clinical and non-clinical supplies, building maintenance, direct and indirect personnel, facility-level training, equipment, and administrative support costs, all at the facility level. We set first-line ARV cost at USD$132 per person per year (2012 USD), as suggested by the MATCH study [28], and second-line ARV cost at $366 (2013 USD) as suggested by the Clinton Health Access Initiative [30]. All ART treatment costs were further increased by 20% to account for above facility programmatic costs incurred by governments and donors [31]. Unit costs for key prevention services were based on regional averages used by the 2011 Investment Framework [31]. All unit costs were converted to 2014 USD using the Consumer Price Indices from the Bureau of Labor Statistics. We discount all future costs and life-years saved at an annual rate of 3%. We determined costs from the health care provider perspective, and did not include medical costs incurred offsite (i.e. inpatient days), patient time, and patient travel costs.

Cost-effectiveness of strategies and scenarios was calculated as the cost in US$ per life-year saved. We applied traditional willingness to pay threshold values in terms of Gross Domestic Product (GDP) per capita income, defining costs per life-year saved of ≤ 1 x GDP per capita income as “highly cost-effective”, and ≤ 3 x per-capita GDP per capita income as “cost-effective”. We chose the lowest country level per capita GDP of all the countries in our analysis as the threshold value (Malawi, 255 US$ in 2014), and also compared estimates to the median (average of Tanzania and Zimbabwe; 943 US$) and highest (South Africa; 6483 US$) per capita GDP of all the countries in our analysis. GDP per capita data were available at: http://data.worldbank.org/indicator/NY.GDP.PCAP.CD

Rapidly scaling up ART treatment access by relaxing supply-side constraints will require upfront investments in health system improvements, such as for training new health care workers and acquiring new facility space. Therefore, we calculated a once-off cost reflecting health system improvements when needed. We first calculated the increased need for health system capacity as the difference between numbers of people on ART in a given year compared to the preceding year. If the difference was larger than zero (i.e. more people on ART), we added an additional per-patient cost of health system investments to account for the extra resources needed for treating these patients. Because of a lack of data, we derived estimates of HIV training and facility costs per patient from National AIDS Spending Assessment (NASA) country reports. For consistency purposes, we used the most up-to-date country report (Zambia, 2012) [32], and assumed investment needs to be similar for the other countries. All unit costs are shown in table S5 of the supplementary material.

1. **Simulations**

We simulated trends in the costs of ART and other prevention services for all countries over the period 2016 to 2050. For all countries, we ran all 6 scenarios with the 40 parameter combinations that provided a good fit to historical epidemiological and ART treatment scale-up data. We calculated the point estimate of each outcome measure as the average over the 40 parameter combinations, and discarded the 1^st^ highest and 1^st^ lowest predictions of the 40 parameter combinations to arrive at a crude 95% interval for parameter uncertainty.

| **Country** | **Fitting period** | **Years of HIV introduction** | **Promiscuity multiplier*** | **FSW multiplier**** |
| --- | --- | --- | --- | --- |
| Ethiopia | 1990 - 1997 | 1987.3 – 1989.9 | 0.41 – 0.72 | 0.75 – 0.84 |
| Kenya | 1990 - 1995 | 1987.8 – 1989.3 | 0.85 – 1.15 | n.a. |
| Malawi | 1990 - 1995 | 1969.3 – 1982.9 | 0.50 – 0.73 | n.a. |
| Mozambique | 1990 - 2003 | 1988.3 – 1990.6 | 0.68 – 0.98 | n.a. |
| Nigeria | 1990 - 2001 | 1983.2 – 1988.9 | 0.20 – 0.69 | 0.75 – 0.93 |
| South Africa | 1990 - 2000 | 1987.9 – 1990.2 | 0.88 – 0.99 | n.a. |
| Tanzania | 1990 - 1994 | 1983.4 – 1987.9 | 0.34 – 0.71 | n.a. |
| Uganda | 1990 - 1990 | 1962.7 – 1981.6 | 0.15 – 0.46 | n.a. |
| Zambia | 1990 - 1995 | 1967.1 – 1984.0 | 0.27 – 0.40 | n.a. |
| Zimbabwe | 1990 - 1994 | 1985.2 – 1987.2 | 1.0 – 1.1 | n.a. |

**Table S2. Fitting period and input parameters for the first phase of the epidemic (growth phase) for all countries.** Ranges reflect the lowest and highest value for each parameter that provided a good fit.

* Promiscuity multiplier is applied to all *personal promiscuity factors* (*p* – see section 1 for explanation).

** FSW multiplier is applied to all individual inclinations to visit a FSW (see section 1 for explanation).

n.a. = not applicable

| **Country** | **Fitting period** | **Start years condom scale-up** | **Condom use end value*** | **ART scale-up fitting period** | **Start years ART scale-up** | **ART scale-up slope**** |
| --- | --- | --- | --- | --- | --- | --- |
| Ethiopia | 1998 - 2013 | 1997.2 – 1999.5 | 71% - 83% | 2004 – 2013 | 2003.2 – 2004.3 | 0.079 – 0.100 |
| Kenya | 1996 - 2013 | 1994.9 – 1996.4 | 71% - 79% | 2004 – 2013 | 2002.2 – 2002.7 | 0.065 – 0.070 |
| Malawi | 1996 - 2013 | 2000.1 – 2002.2 | 51% - 65% | 2004 – 2013 | 2003.5 – 2004.2 | 0.081 – 0.101 |
| Mozambique | 2004 - 2013 | 2001.1 – 2005.8 | 34% - 56% | 2004 – 2013 | 2001.7 – 2003.9 | 0.042 – 0.072 |
| Nigeria | 2002 - 2013 | 2001.0 – 2003.9 | 33% - 46% | 2004 – 2013 | 2002.3 – 2003.7 | 0.016 – 0.022 |
| South Africa | 2001 - 2013 | 2000.0 – 2001.5 | 49% - 58% | 2004 – 2013 | 2002.6 – 2004.2 | 0.061 – 0.093 |
| Tanzania | 1995 - 2013 | 1994.8 – 1996.9 | 38% - 59% | 2004 – 2013 | 2003.6 – 2004.4 | 0.062 – 0.097 |
| Uganda | 1991 - 2013 | 1992.1 – 1996.4 | 29% - 46% | 2004 – 2013 | 2000.7 – 2002.4 | 0.055 – 0.079 |
| Zambia | 1996 - 2013 | 1994.3 – 1997.2 | 23% - 35% | 2004 – 2013 | 2002.3 – 2003.2 | 0.103 – 0.134 |
| Zimbabwe | 1995 - 2013 | 1994.4 – 1996.4 | 82% - 89% | 2004 – 2013 | 2002.1 – 2003.1 | 0.083 – 0.098 |

**Table S3. Fitting period and input parameters for the second phase of the epidemic (levelling off/decline phase) for all countries.** Ranges reflect the lowest and highest value for each parameter that provided a good fit.

* Only reflects condom use in casual relationships and commercial sex

** Modelled as the fraction of the population that has access to HIV treatment and care. Slope represents the annual increase in access to care.

|  | **Scenarios 1 – 5: liability, health system constraints, continued scale-up, rapid scale-up, and 90-90-90** | | |
| --- | --- | --- | --- |
|  | *PMTCT* | *Circumcision* | *Condom distribution** |
| Ethiopia | 55% | 76% | 71% - 83% |
| Kenya | 63% | 84% | 71% - 79% |
| Malawi | 79% | 27% | 51% - 65% |
| Mozambique | 84% | 56% | 34% - 56% |
| Nigeria | 27% | 81% | 33% - 46% |
| South Africa | 90% | 35% | 49% - 58% |
| Tanzania | 73% | 70% | 38% - 59% |
| Uganda | 75% | 25% | 29% - 46% |
| Zambia | 76% | 12% | 23% - 35% |
| Zimbabwe | 78% | 10% | 82% - 89% |
|  |  |  |  |
|  | **Scenario 6: 90-90-90+ (i.e. expanded 90-90-90)** | | |
|  | *PMTCT* | *Circumcision* | *Condom distribution* |
| Ethiopia | 95% | 80% | 88% - 93% |
| Kenya | 95% | 84% | 88% - 92% |
| Malawi | 95% | 80% | 80% - 86% |
| Mozambique | 95% | 80% | 74% - 82% |
| Nigeria | 95% | 81% | 73% - 78% |
| South Africa | 95% | 80% | 80% - 83% |
| Tanzania | 95% | 80% | 75% - 84% |
| Uganda | 95% | 80% | 72% - 78% |
| Zambia | 95% | 80% | 69% - 74% |
| Zimbabwe | 95% | 80% | 93% - 96% |

**Table S4. Coverage of prevention interventions for all scenarios.** Coverage of PMTCT and circumcision derived from UNAIDS reported data [20]

* Reflects condom use in casual relationships and commercial sex

|  | ART costs (per year)* | Programmatic costs (per year) | Counseling and testing (per test) | Health system investment** | VMMC (per patient) | ANC screening (per test) | PMTCT (per pregnancy) | Condom distribution (per condom) | Behavior change program*** |
| --- | --- | --- | --- | --- | --- | --- | --- | --- | --- |
| Ethiopia | 330.15 | 66.03 | 16.36 | 150.4 | 63.01 | 4.39 | 667.70 | 0.20 | 22.26 |
| Kenya | 416.14 | 83.23 | 16.36 | 150.4 | 63.01 | 4.39 | 667.70 | 0.20 | 22.26 |
| Malawi | 232.09 | 46.42 | 16.36 | 150.4 | 63.01 | 4.39 | 667.70 | 0.20 | 22.26 |
| Mozambique | 351.84 | 70.37 | 16.36 | 150.4 | 63.01 | 4.39 | 667.70 | 0.20 | 22.26 |
| Nigeria | 553.75 | 110.75 | 16.36 | 150.4 | 63.01 | 4.39 | 667.70 | 0.20 | 22.26 |
| South Africa | 651.94 | 130.39 | 16.36 | 150.4 | 63.01 | 4.39 | 667.70 | 0.20 | 22.26 |
| Tanzania | 371.55 | 74.31 | 16.36 | 150.4 | 63.01 | 4.39 | 667.70 | 0.20 | 22.26 |
| Uganda | 347.29 | 69.46 | 16.36 | 150.4 | 63.01 | 4.39 | 667.70 | 0.20 | 22.26 |
| Zambia | 470.54 | 94.11 | 16.36 | 150.4 | 63.01 | 4.39 | 667.70 | 0.20 | 22.26 |
| Zimbabwe | 404.40 | 80.88 | 16.36 | 150.4 | 63.01 | 4.39 | 667.70 | 0.20 | 22.26 |

**Table S5. Unit costs of HIV treatment and prevention interventions.** Costs are in US$, sources are described in section 3 of the supplementary material. *ART costs are the costs for first line and second line drugs combined, assuming 10% of patients is on second line treatment. **Health system investments costs are only counted when the number of patients on treatment in a certain year exceeds that of the previous year. Costs are calculated by multiplying the unit costs with the difference in patient numbers between the years. ***Costs of the behavior change program are per 15-24 year old adult. We assume a 10% increase in costs to reflect further reductions in partner change rates in the 90-90-90+ scenario

**Table S6 Sensitivity analysis of the impact of health seeking rates on the cost-effectiveness of ART at any CD4 cell count versus ART at CD4 cell counts of ≤500 cells/µL**

|  |  | **Costs per life year saved (US$)**  **[range]** | | |  |
| --- | --- | --- | --- | --- | --- |
|  | *Baseline* | | *20% lower health seeking* | *20% higher health seeking* | |
| **ART at any CD4 count vs. ART at CD4 ≤500 cells/μL** |  | |  |  | |
| 2. Health systems constraints | 54 [51; 57] | | 85 [80; 92] | 44 [42; 47] | |
| 3. Continued scale-up | 208 [191; 227] | | 200 [184; 219] | 215 [199; 235] | |
| 4. Rapid scale-up | 114 [106; 124] | | 101 [93; 110] | 128 [119; 140] | |
| 5. 90-90-90 | 219 [203; 237] | | 225 [209; 243] | 213 [198; 232] | |
| 6. 90-90-90+ | 1358 [1276; 1435] | | 1305 [1223; 1379] | 1414 [1330; 1494] | |

**Captions to supplementary figures**

**Figure S1. HIV prevalence simulated by STDSIM compared to UNAIDS estimates for the 10 countries in the analysis over the period 1990 – 2014.** Model average reflects the average prediction form 40 unique parameter combinations. The 95% interval reflects the uncertainty in these parameters (see section 2.3 of the supplementary material). Data from UNAIDS.[33]

**Figure S2. ART coverage simulated by STDSIM compared to UNAIDS estimates for the 10 countries in the analysis over the period 1990 – 2014.** Model average reflects the average prediction form 40 unique parameter combinations. The 95% interval reflects the uncertainty in these parameters (see section 2.3 of the supplementary material). Data from UNAIDS.[33]

1. **References**

1. Hontelez J, de Vlas S, Tanser F, Bakker R, Barnighausen T, Newell M*, et al.* The Impact of the New WHO Antiretroviral Treatment Guidelines on HIV Epidemic Dynamics and Cost in South Africa. *PLoS One* 2011,**6**:e21919.

2. Korenromp EL, Van Vliet C, Bakker R, De Vlas SJ, Habbema JD. HIV spread and partnership reduction for different patterns of sexual behavior – a study with the microsimulation model STDSIM. *Mathematics and Population Studies* 2000,**8**:135-173.

3. Orroth KK, Freeman EE, Bakker R, Buve A, Glynn JR, Boily MC*, et al.* Understanding the differences between contrasting HIV epidemics in east and west Africa: results from a simulation model of the Four Cities Study. *Sex Transm Infect* 2007,**83 Suppl 1**:i5-16.

4. van der Ploeg CPB, Van Vliet C, De Vlas SJ, Ndinya-Achola JO, Fransen L, van Oortmarssen GJ*, et al.* STDSIM: A Microsimulation Model for Decision Support in STD Control. *Interfaces* 1998,**28**:84-100.

5. Ghys PD, Zaba B, Prins M. Survival and mortality of people infected with HIV in low and middle income countries: results from the extended ALPHA network. *Aids* 2007,**21 Suppl 6**:S1-4.

6. Attia S, Egger M, Muller M, Zwahlen M, Low N. Sexual transmission of HIV according to viral load and antiretroviral therapy: systematic review and meta-analysis. *AIDS* 2009,**23**:1397-1404.

7. Johnson LF, Mossong J, Dorrington RE, Schomaker M, Hoffmann CJ, Keiser O*, et al.* Life expectancies of South African adults starting antiretroviral treatment: collaborative analysis of cohort studies. *PLoS Med* 2013,**10**:e1001418.

8. Walensky RP, Wolf LL, Wood R, Fofana MO, Freedberg KA, Martinson NA*, et al.* When to start antiretroviral therapy in resource-limited settings. *Ann Intern Med* 2009,**151**:157-166.

9. Badri M, Bekker LG, Orrell C, Pitt J, Cilliers F, Wood R. Initiating highly active antiretroviral therapy in sub-Saharan Africa: an assessment of the revised World Health Organization scaling-up guidelines. *AIDS* 2004,**18**:1159-1168.

10. UNDP. International Human Development Indicators. In: United Nations Development Programme; 2010.

11. Hontelez JA, Lurie MN, Barnighausen T, Bakker R, Baltussen R, Tanser F*, et al.* Elimination of HIV in South Africa through expanded access to antiretroviral therapy: a model comparison study. *PLoS Med* 2013,**10**:e1001534.

12. Korenromp EL, Bakker R, De Vlas SJ, Robinson NJ, Hayes R, Habbema JD. Can behavior change explain increases in the proportion of genital ulcers attributable to herpes in sub-Saharan Africa? A simulation modeling study. *Sex Transm Dis* 2002,**29**:228-238.

13. Hontelez JA, Lurie MN, Newell ML, Bakker R, Tanser F, Bärnighausen T*, et al.* Ageing with HIV in South Africa. *AIDS* 2011,**25**:1665-1667.

14. Hontelez JAC, De Vlas SJ, Baltussen R, Newell M, Bakker R, Tanser F*, et al.* The impact of antiretroviral treatment on the age composition of the HIV epidemic in sub-Saharan Africa. *AIDS* 2012,**26 (Suppl 1)**:S19 - S30.

15. WHO. Life tables for WHO member States. In. Geneva: World Health Organization; 2011.

16. WHO. The global burden of disease 2004; Update (2008). In. Geneva: World Health Organization; 2011.

17. UN. World Fertility Data 2008. In. Geneva: United Nations Population Division - Fertility and Family Planning Section; 2008.

18. Williams BG, Lloyd-Smith JO, Gouws E, Hankins C, Getz WM, Hargrove J*, et al.* The potential impact of male circumcision on HIV in Sub-Saharan Africa. *PLoS Med* 2006,**3**:e262.

19. UNAIDS. Report on the Global AIDS epidemic 2010. In. Geneva: UNAIDS; 2010.

20. UNAIDS. AIDSInfo Online Database. In; 2014.

21. UN. World Population Prospects, the 2010 revision. In. Geneva: United Nations Population Division; 2010.

22. Hontelez JA, de Vlas SJ, Tanser F, Bakker R, Bärnighausen T, Newell ML*, et al.* The impact of the new WHO antiretroviral treatment guidelines on HIV epidemic dynamics and cost in South Africa. *PLoS One* 2011,**6**:e21919.

23. Cleland J, Ali MM, Shah I. Trends in protective behaviour among single vs. married young women in sub-Saharan Africa: the big picture. *Reprod Health Matters* 2006,**14**:17-22.

24. Rehle TM, Hallett TB, Shisana O, Pillay-van Wyk V, Zuma K, Carrara H*, et al.* A decline in new HIV infections in South Africa: estimating HIV incidence from three national HIV surveys in 2002, 2005 and 2008. *PLoS One* 2010,**5**:e11094.

25. Johnson LF, Hallett TB, Rehle TM, Dorrington RE. The effect of changes in condom usage and antiretroviral treatment coverage on human immunodeficiency virus incidence in South Africa: a model-based analysis. *J R Soc Interface* 2012,**9**:1544-1554.

26. Patel P, Borkowf CB, Brooks JT, Lasry A, Lansky A, Mermin J. Estimating per-act HIV transmission risk: a systematic review. *AIDS* 2014,**28**:1509-1519.

27. Becquet R, Ekouevi DK, Arrive E, Stringer JS, Meda N, Chaix ML*, et al.* Universal antiretroviral therapy for pregnant and breast-feeding HIV-1-infected women: towards the elimination of mother-to-child transmission of HIV-1 in resource-limited settings. *Clin Infect Dis* 2009,**49**:1936-1945.

28. Tagar E, Sundaram M, Condliffe K, Matatiyo B, Chimbwandira F, Chilima B, et al. Multi-country analysis of treatment costs for HIV/AIDS (MATCH): facility-level ART unit cost analysis in Ethiopia, Malawi, Rwanda, South Africa and Zambia. PLoS One 2014,9:e108304.

29. Menzies NA, Berruti AA, Berzon R, Filler S, Ferris R, Ellerbrock TV, et al. The cost of providing comprehensive HIV treatment in PEPFAR-supported programs. AIDS 2011,25:1753-1760.

30. Clinton Health Access Initiative. ARV market report: the state of the antiretroviral drug market in low-and middle-income countries, 2014-2019. Available from: http://www.clintonhealthaccess.org/content/uploads/2015/11/CHAI-ARV-Market-Report-2015_FINAL.pdf

31. Schwartlander B, Stover J, Hallett T, Atun R, Avila C, Gouws E, et al. Towards an improved investment approach for an effective response to HIV/AIDS. Lancet 2011,377:2031-2041.

32. UNAIDS. Zambia's National AIDS spending assessment 2012. Accessed from <http://www.unaids.org/en/dataanalysis/knowyourresponse/nasacountryreports32>.

33. UNAIDS. AIDSinfo Online Database. In.
